# Mandibular advancement devices vs nasal-continuous positive airway pressure in the treatment of obstructive sleep apnoea. Systematic review and meta-analysis

**DOI:** 10.4317/medoral.21671

**Published:** 2017-06-04

**Authors:** Giovanni Cammaroto, Cosimo Galletti, Francesco Galletti, Bruno Galletti, Claudio Galletti, Cosme Gay-Escoda

**Affiliations:** 1 MD. Department of Otorhinolaryngology, University of Messina, Messina, Italy; 2 DDS, MS. Professor of Integrated Adult Dentistry. School of Dentistry, University of Barcelona. Researcher of the IDIBELL Institute; 3 MD, PhD. Department of Otorhinolaryngology, University of Messina, Messina, Italy; 4 MD, Department of Anaesthesiology, University of Messina, Messina, Italy; 5 MD, DDS, PhD, MS, EBOS, OMFS. Chairman and Professor of the Oral and Maxillofacial Surgery Department. School of Dentistry, University of Barcelona. Coordinator/Researcher of the IDIBELL Institute. Head of Oral and Maxillofacial Surgery, Department of the Teknon Medical Center, Barcelona, Spain

## Abstract

**Background:**

Obstructive sleep apnoea (OSA) is a common disorder that may affect at least 2 to 4% of the adult population. Nasal-Continuous Positive Airway Pressure (N-CPAP) is today considered the gold standard for the treatment of OSA. The development of oral appliances (OAs) represents a new approach for the management of this pathology. The aim of this systematic review is to compare the efficacy of OAs and N-CPAP in the treatment of patients with mild to severe OSA.

**Material and Methods:**

A PubMed-MEDLINE and Cochrane databases search of articles published between 1982 and 2016 comparing the effect of N-CPAP and OAs in OSA patients was conducted during July 2016. The studies were selected and stratified according to PRISMA and SORT criteria. The main outcome measure was post-treatment Apnoea-Hypopnoea Index (AHI) while secondary outcomes included post-treatment Epworth Score Scale (ESS) score and lowest Oxygen Saturation level.

**Results:**

N-CPAP was significantly more effective in suppressing AHI than OA. Moreover, N- CPAP was significantly more effective in increasing post-treatment lowest Oxygen Saturation level than OA. However, no significant different in decreasing ESS values was found between the two treatments.

**Conclusions:**

On the basis of evidence in this review it would appear appropriate to offer OA therapy to those who are unwilling or unable to persist with CPAP therapy. N-CPAP still must be considered the gold standard treatment for OSA and, therefore, OAs may be included in the list of alternative options.

** Key words:**CPAP, obstructive sleep apnoea, oral appliances.

## Introduction

Obstructive Sleep Apnoea (OSA) is a common disorder that may affect at least 2 to 4% of the adult population (1). The management of OSA depends on the severity of symptoms and aetiology of airway obstruction. Among the treatment options there are some conservative measures such as weight reduction, relief of nasal obstruction and avoidance of alcohol. However, the use of Nasal-Continuous Positive Airway Pressure (N-CPAP), which is considered the gold standard for the treatment of OSA, is necessary in the majority of cases (2). N-CPAP is effective in treating OSA by “splinting” the airway via creation of a positive pressure applied through the nares (3).

Recently, the scientific community has been orientating towards alternatives to N-CPAP considering the low adherence to treatment and the patients’ low level of satisfaction.

Today Oral Appliances (OAs) and several surgical techniques can be considered satisfactory options for OSA treatment.

The development of OAs represents a new approach for the management of OSA (4). An OA is a device that fits within the oral cavity and prevents upper airway collapse occurring in OSA patients by advancing the mandible and, therefore, increasing the upper airways diameters. For patients with severe OSA, a trial of N-CPAP is required prior to their use, and surgery may be preferred over an OA for N-CPAP failures. Moreover, predicting which patients will have successful OA titration and treatment response is still difficulty (5).

A recent American Academy of Sleep Medicine (AASM) guideline concluded that OAs are less effective than N-CPAP but are a reasonable alternative for patients with mild to moderate OSA (6).

However, the studies used to establish these guidelines are limited by small sample sizes, select patient populations, the absence of device titration during polysomnography and there are few data concerning the comparison between N-CPAP and OA in patients with OSA.

The aim of this systematic review is to compare the efficacy of OA and N-CPAP in the treatment of patients with mild to severe OSA.

## Materials and Methods

-Literature search protocol 

A PubMed-MEDLINE and Cochrane databases search of articles published between 1982 and 2016 was conducted during July 2016. In an initial search, the terms “oral appliance”, “nasal-continuous positive airway pressure”, “obstructive sleep apnoea” were used. Search was limited to human studies, and articles written in English. The terms were then merged in a second search, using the Boolean operator “AND”, in order to obtain the articles that included two or more of the used search terms. Items founds were analysed to verify the relevance of these in relation to the topic under study. The irrelevant articles were discarded.

The PRISMA criteria were used to select the studies (7) 

The inclusion criteria were: Randomized controlled trials (RCT) and RCT crossover comparing N-CPAP to Mandibular Advancement Devices (MADs), patients affected by mild to severe OSA, patients who were not treated previously, studies reporting pre-treatment and post-treatment Apnoea-Hypopnoea Index (AHI) values.

Next, the items were stratified according to their level of scientific evidence, using the PRISMA criteria. Finally, the SORT criteria (Strength of Recommendation Taxonomy) ( Table 1, Table 2) were used to check the level of scientific evidence of the included studies (8). Subsequently, according to the level of scientific evidence of the reviewed articles, a recommendation level was declared in favour of, or against the OA or N-CPAP.

**Table 1:**
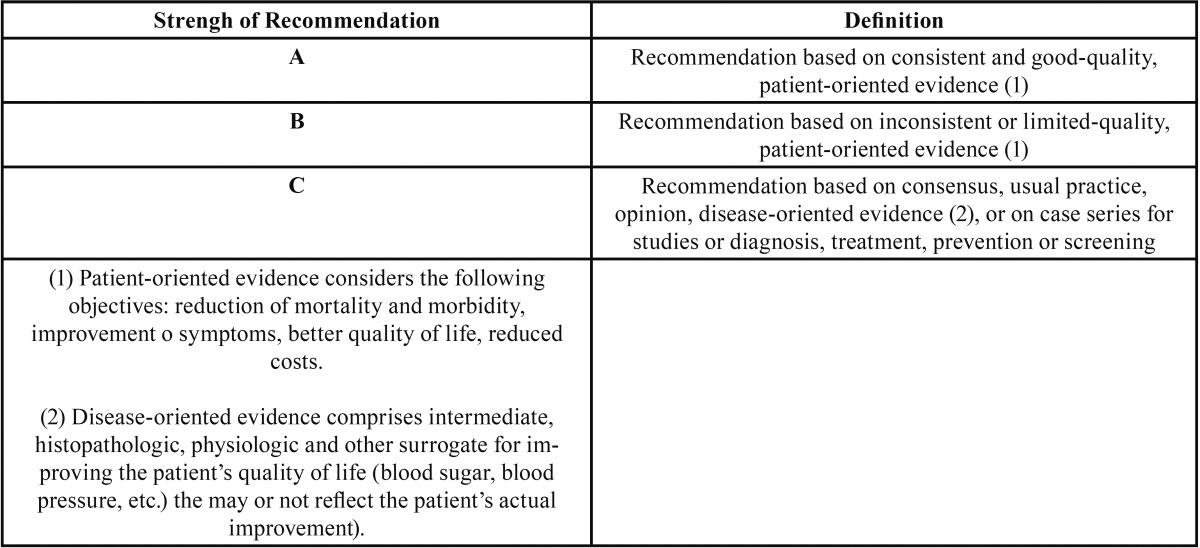
SORT Criteria (Strength of Recommendation Taxonomy) (8).

**Table 2:**
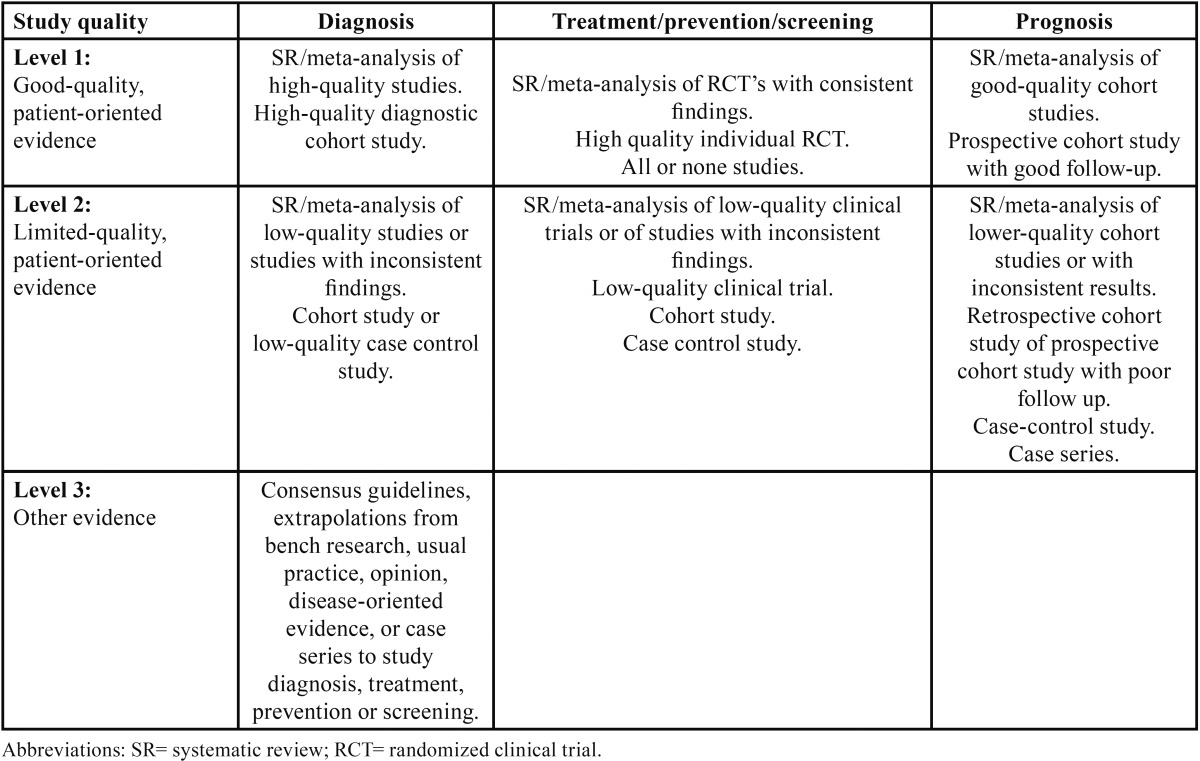
Levels of scientific evidence (8).

-Outcome measurements

The primary outcome was the post-treatment AHI value.

The secondary outcomes were the post-treatment Epworth Score Scale (ESS) score and lowest Oxygen Saturation level.

AHI and lowest Oxygen Saturation level are two objective outcomes extracted from the polysomnography, which is the most important diagnostic tool for the evaluation of patients affected by OSA. On the other hand, ESS score is a subjective outcome obtained from a questionnaire used to measure the daytime sleepiness.

-Analysis protocol

Data from the studies were first extracted and assessed by the principal investigator (GC), and thereafter independently by the co-author (CG) using standardized data forms. Articles were examined for data resolution with the intent to perform a meta-analysis.

Data extraction included the following items:

1. Population: age, number of patients studied, and treatment duration.

2. Intervention: OA (type MAD)

3. Control: N-CPAP

4. Outcomes: as above

5. Design: method of randomisation and allocation concealment

-Assessment of risk of bias in included studies

Different methods of meta-analyses were considered in reviewing the literature to seek results that would provide meaningful analysis with the least risk of introducing biases. The Quality Assessment of Studies (QUADAS-2) tool was used to evaluate relevant study design characteristics of the included studies. This type of quality assessment was designed in 2003 and updated in 2011 to help judge the risks of bias and applicability (9).

-Statistical analysis

Random effect models were used to generate pooled estimates. Data were analysed using generic inverse radiance method and *p*<0.05 is regarded as statistically significant.

Combined summary statistics of the standardized (STD) paired difference in mean for the individual studies are shown. Combined STD paired differences in means were calculated and a two-sided *p* value<0.05 was considered to indicate statistical significance. A 2-based test of homogeneity was performed and the inconsistency index (I2) statistic was determined. If I2 was 50 or 75 %, the studies were considered to be heterogeneous or highly heterogeneous, respectively. If I2 was below 25 %, the studies were considered to be homogeneous. If the I2 statistic (>50 %) indicated that heterogeneity existed between studies, a random-effects model was calculated.

We have used the different comparisons to display the results. Subgroup analysis by severity of OSA was not possible due to the heterogeneous populations recruited in the studies.

Data from crossover studies have been analysed as parallel and crossover group data as the trialists made first arm data available upon request.

Publication bias was also tested using the funnel plot. A funnel plot is a type of scatter plot that can be useful to understand study heterogeneity of meta-analysis. The funnel plot examines the sample size on the y axis (plotted as the standard error of the log odds ratio) and treatment effect on the x -axis (plotted as the odds ratio).

Analyses were performed with RevMan (Nordic Cochrane Center, Copenhagen, Denmark).

## Results

Following the initial analysis, a total of 6 articles were selected and included in the study. These items were stratified by level of scientific evidence, using the SORT criteria (8). All the articles showed a level of evidence 2 and strenght of recommendation B.

**Figure 1:**
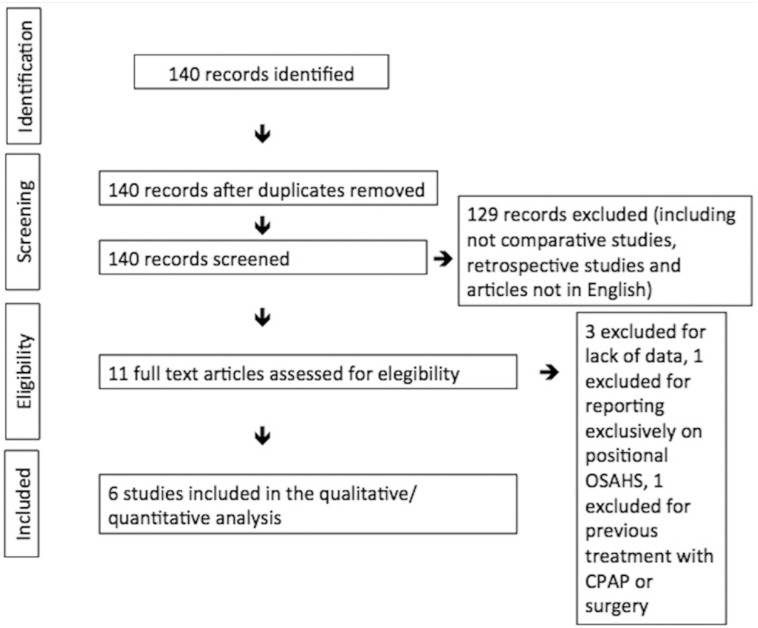
PRISMA diagram showing the systematic review process.

**Figure 2:**
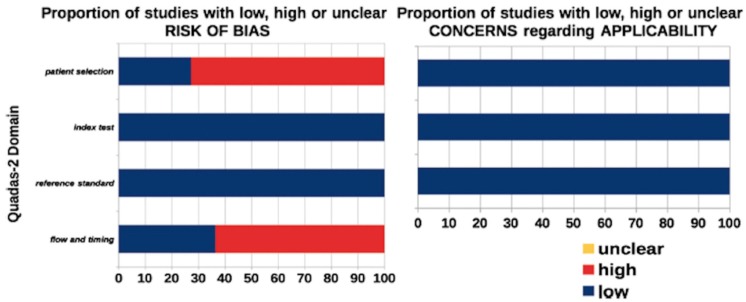
Graphical display of QUADAS-2.

The search was performed in July 2016 and yielded 140 articles, of which six articles met inclusion’s criteria (2;10-14) (Fig. 1). The graphical display of QUADAS-2 shows that while the applicability of these studies is very high, there is a risk on bias when considering patient selection and the flow of the studies (Fig. 2).

 Table 3 and  Table 4 show data about the type of study, year of publication, number of publication, treatment duration, age, body mass index (BMI), pre-treatment AHI, lowest Oxygen Saturation level and ESS. Four studies were RCT-crossover while two were RCT. Ferguson and Barnes report on longer treatment duration in comparison with the other studies (≥6 months).

**Table 3:**
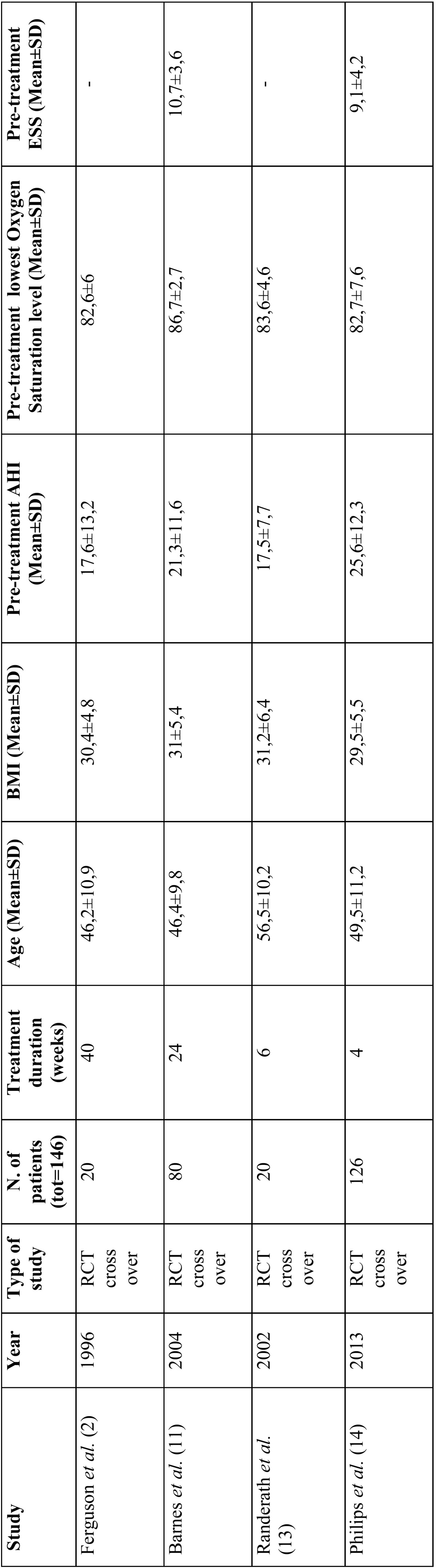
Data of included Randomized Clinical Trials cross-over (BMI=Body Mass Index; AHI= Apnoea-Hypopnoea Index; ESS= Epworth Score Scale).

**Table 4:**
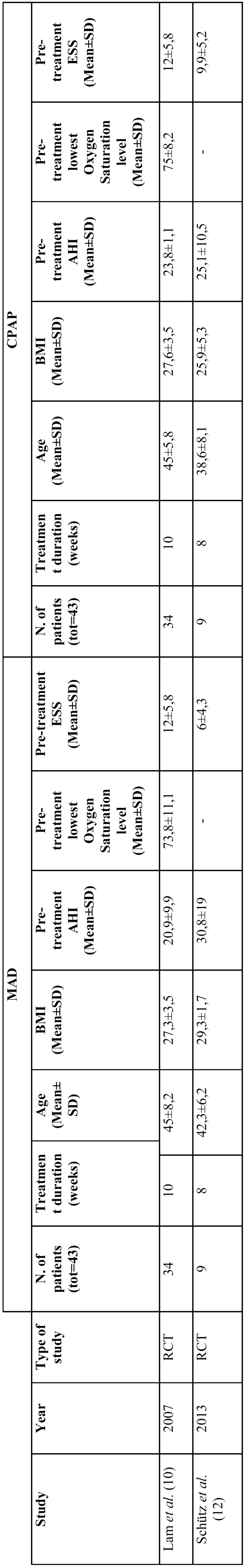
Data of included Randomized Clinical Trials (BMI=Body Mass Index; AHI= Apnoea-Hypopnoea Index; ESS= Epworth Score Scale).

AHI

**Figure 3:**
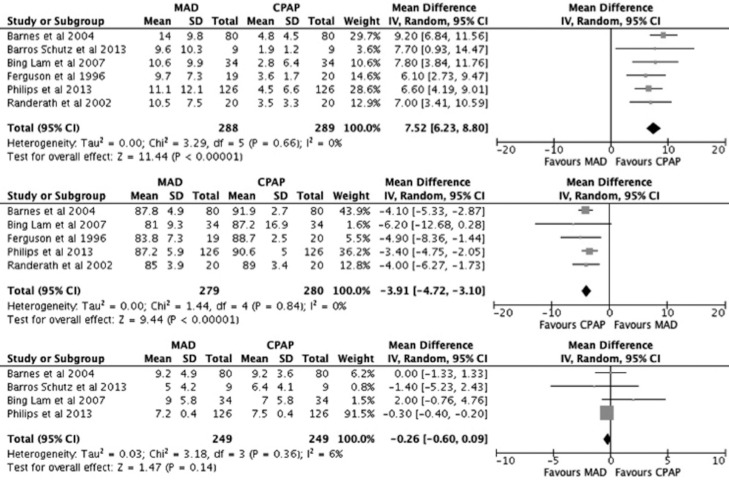
Forest plot comparing the effect of N-CPAP and Mandibular Advancement Device (MAD) on the post-treatment Apnoea-Hypopnoea Index (AHI) (top). Forest plot comparing the effect of CPAP and MAD on the post-treatment lowest Oxygen Saturation level (middle). Forest plot comparing the effect of CPAP and MAD on the post-treatment Epworth Score Scale (ESS) (bottom).

CPAP was significantly more effective in suppressing AHI than MAD. No heterogeneity was highlighted in this analysis (Fig. 3).

Lowest Oxygen Saturation level

CPAP was significantly more effective in increasing post-treatment lowest Oxygen Saturation level than MAD. No heterogeneity was highlighted in this analysis (Fig. 3).

ESS

No significant different in decreasing ESS values was found between the two treatments. A low grade of heterogeneity was registered. However, MADs seem to guarantee slightly better outcomes (Fig. 3).

## Discussion

Today, N-CPAP is considered the gold standard treatment for OSA patients, being the most evidence based supported therapeutic option in the scientific literature. Other treatments such as surgery and OAs have been subsequently introduced in the armamentarium of sleep medicine but higher evidence is needed to confirm the promising results presented by many authors.

In this study, meta-analysis of the outcomes of three items (AHI, ESS, lowest Oxygen Saturation level) was performed to compare the effectiveness of MADs with N-CPAP for the treatment of OSA patients.

Although the number of eligible studies was low, these were RCT studies of high-quality evidence. However, the blinding of patients or assessors was impossible considering the different shape of OAs and N-CPAP. Thus, blinding was absent in this study decreasing the evidence grade.

Taking account that four of the included studies were RCT crossover and that the duration of treatment was different among the studies the following can be highlighted.

As a result of the analysis, a more significant decrease in AHI was observed in patients treated with N-CPAP. Moreover, N-CPAP increased lowest Oxygen Saturation level more significantly than OAs.

Finally, although the ESS score was lower after OA therapy in comparison with N-CPAP, no significant difference was observed.

The results of our study were almost similar to those of other reviews underlying the role of N-CPAP as first choice treatment for OSA patients (15,16).

Despite the better results obtained with N-CPAP, other aspects deserve attention.

For instance, a study on the degree of patient satisfaction showed better outcomes in patients treated with OAs (17). Other studies compared the frequency of use between OA and CPAP and showed that the OA was used for a longer time (10,17).

Adherence to treatment has always been a main issue for N-CPAP therapy.

Evidence suggests that use of CPAP for longer than 6 hours decreases sleepiness, improves daily functioning, and restores me-mory to normal levels (18).

For this reason, the scientific community has been recently focusing on treatment alternatives such as OAs and surgeries, which are preferred by patients and, in case of a surgical choice, do not present problems of adherence.

In the studies included in a Cochrane meta-analysis, various types of OAs were used, such as mandibular advancement devices, tongue-retaining devices and those that allowed mouth opening and limited mouth-opening devices (15). On the other hand, our study focused on the comparison between MADs and N-CPAP, this allowing a more homogeneous analysis.

Various studies have been conducted on the shape of the OA. Regarding this aspect, MADs were reported to be more effective than tongue-retaining devices (TRD) (19). Other studies have shown that OAs allowing titratable mandibular advancement and preventing mouth opening may be more effective for the treatment of OSA (20,21).

The cost of OAs and their side-effects/complications also need to be mentioned.

Both treatment options may lead to different adverse effects (2,13). Jaw and oral pain generally occur more frequently with OA than with N-CPAP (15). Other studies reported higher rates of excessive salivation and appliance removal during sleep with OA, but higher rates of leak, dry upper airway, stuffy nose, and inconvenience with N-CPAP (15).

Unfortunately, data on long-term complications and costs in patients treated with OAs and N-CPAP are limited. Furthermore, cost-effectiveness analysis may be needed to support the role of OAs as an alternative to N-CPAP for OSA patients.

Finally, from the current scientific evidence it seems that the treatment of OSA needs to be tailored for each patients. For instance, patients with nasal obstruction caused by septal deviation or poliposis may not benefit from the use of N-CPAP while patients affected by disorder of the temporo-mandibular joint may worsen when using OAs.

A holistic evaluation of OSA patients is mandatory before choosing the most appropriate treatment: upper airways anatomy, sleep physiology and metabolic status need to be assessed in detail in order to select the best candidate, especially when alternatives to N-CPAP are taken into consideration.

On the basis of evidence in this review it would appear appropriate to offer OA therapy to those who are unwilling or unable to persist with N-CPAP therapy. N-CPAP still must be considered the gold standard treatment for OSA and, therefore, OAs may be included in the list of alternative options. This recommendation is drawn from evidence of limited duration. Long-term effects of these two treatments are not currently evaluable.
